# Understanding Central Mechanisms of Acupuncture Analgesia Using Dynamic Quantitative Sensory Testing: A Review

**DOI:** 10.1155/2013/187182

**Published:** 2013-05-12

**Authors:** Jiang-Ti Kong, Rosa N. Schnyer, Kevin A. Johnson, Sean Mackey

**Affiliations:** ^1^Stanford Systems Neuroscience & Pain Laboratory, Department of Anesthesiology, Division of Pain Medicine, School of Medicine, Stanford University, 1070 Arastradero Road, Suite 200, Palo Alto, CA 94304, USA; ^2^School of Nursing, The University of Texas at Austin, Austin, TX 78701, USA

## Abstract

We discuss the emerging translational tools for the study of acupuncture analgesia with a focus on psychophysical methods. The gap between animal mechanistic studies and human clinical trials of acupuncture analgesia calls for effective translational tools that bridge neurophysiological data with meaningful clinical outcomes. Temporal summation (TS) and conditioned pain modulation (CPM) are two promising tools yet to be widely utilized. These psychophysical measures capture the state of the ascending facilitation and the descending inhibition of nociceptive transmission, respectively. We review the basic concepts and current methodologies underlying these measures in clinical pain research, and illustrate their application to research on acupuncture analgesia. Finally, we highlight the strengths and limitations of these research methods and make recommendations on future directions. The appropriate addition of TS and CPM to our current research armamentarium will facilitate our efforts to elucidate the central analgesic mechanisms of acupuncture in clinical populations.

## 1. Overview of Research on Acupuncture Analgesia

The treatment of chronic pain is the most well-known clinical application of acupuncture in the west [1, 2]. Acupuncture originated in China more than 2000 years ago and has gained popularity in America since the landmark NIH Consensus Conference in 1997 [3]. Despite broad use, there continues to be ambiguity regarding the efficacy and mechanisms of acupuncture as an analgesic modality. Discrepancies between the results of basic science experiments and clinical trials of acupuncture underscore the controversy surrounding its therapeutic value. The purpose of this review is to outline emerging translational clinical research methods for assessing the central mechanisms of acupuncture analgesia in humans. We begin by summarizing our current understanding of the analgesic mechanisms of acupuncture based on animal and human clinical studies.

### 1.1. Animal Studies

Animal studies have identified many potential biochemical and neuroanatomical substrates of acupuncture analgesia. Wang et al. [4, 5], Zhao [6], and Han [7, 8] have published excellent comprehensive reviews of these studies. From a biochemical standpoint, it appears that acupuncture may alter the metabolism of substrates involved in both the ascending facilitory pathways (N-methyl-D-aspartate receptors [9], substance P [10], and interleukin-1 [11]) and the descending inhibitory pain pathways (endogenous opioids [7], serotonin [12], and norepinephrine [13]). From a neuroanatomical standpoint, several central nervous system structures are reported to mediate acupuncture analgesia, including the periaqueductal gray, the nucleus raphe magnus, the locus ceruleus, the arcuate nucleus, the amygdala, and the nucleus accumbens [4, 6]. It is important to note the link between the biochemical and anatomical substrates. For example, low-frequency electroacupuncture triggers the release of enkephalins and endorphins in the periaqueductal gray, the arcuate nucleus, and the caudate nucleus [14]. These structures then send projections to the spinal dorsal horn via the dorsal lateral funiculi [15]. Increases in serotonin release at the nucleus raphe magnus and norepinephrine release in the locus ceruleus are also crucial to analgesia induced by electroacupuncture [13].

In addition to the classic neurotransmitters and anatomical pathways involved in central pain processing, other mechanisms also contribute to acupuncture analgesia [6], including the hypothalamus-pituitary-adrenal axis (regulating peripheral inflammatory response to pain) [16], the autonomic nervous system (regulating local circulation) [17, 18], and the glial system [19] (contributing to inflammation around spinal and cerebral neural pathways). 

### 1.2. Human Studies

Although animal studies can provide insight into acupuncture's mechanism of action, establishing the efficacy of acupuncture for treating chronic pain in humans is challenging, owing to the variability of study methods and outcomes [20]. However, an increasing body of robust and rigorous evidence indicates that acupuncture may be an effective intervention for the management of chronic pain [21–23]. Researchers from the Acupuncture Trialists' Collaboration, a group, which was established to synthesize data from high-quality randomized trials on acupuncture for chronic pain, recently published a meta-analysis of 29 clinical trials involving 17,922 patients [23]. The analysis showed that acupuncture consistently yielded greater pain reduction as compared with controls in back and neck pain, arthritis, and headaches. When sham acupuncture was used as the control, the differences were modest but remained statistically significant. Larger differences were seen when standard care (which typically included oral medications and regular physician and physical therapy visits) was used as the control [23].

These results indicate that both specific (i.e., site of needling and stimulation techniques) and nonspecific (i.e., context effects, expectations, etc.) components can contribute to acupuncture's therapeutic effect in treating chronic pain. Understanding the possible mechanisms of these effects in chronic pain remains crucial in elucidating the potential therapeutic value of acupuncture in chronic pain. 

### 1.3. Need for Translational Studies Bridging Mechanisms Observed in Animals to Clinical Populations

Animal studies are of limited benefit in fully modeling the human experience of acupuncture and of chronic pain, and the research methods used in clinical trials provide only a limited understanding of acupuncture's mechanism of action in humans. This gap between animal mechanistic studies and human clinical trials remains one of the greatest challenges in acupuncture research today. The white paper published by the Society of Acupuncture Research (SAR) acknowledged this challenge and proposed goals for future studies [24]. One of the key recommendations was the development of biomarkers that can provide meaningful correlations between physiological effects measured in animal studies and patient-reported outcomes in clinical trials.

To this end, we discuss the emerging translational research methods for assessing the central mechanisms of action of acupuncture analgesia in humans. As background, we first review the basic mechanisms of the central nervous system involved in nociception and human pain perception. Next, we focus the review on two research approaches that likely will emerge as valuable tools for understanding pain processing in acupuncture: temporal summation and conditioned pain modulation. We describe the physiologic mechanism, methodology, and applications of these methods in pain research. Then, we examine the current application of temporal summation and conditioned pain modulation to acupuncture research and make recommendations on future directions.

## 2. Nociceptive Pathways and Neural Processing

### 2.1. Nociceptive Pathways

Five major components are involved in the perception of pain: (1) the primary peripheral nociceptors; (2) the spinal secondary neurons; (3) the relay neurons (such as those in the thalamus); (4) cortical and subcortical networks responsible for sensory, emotional, and cognitive integration of pain (e.g., the primary sensory cortex, insula, prefrontal cortex, and anterior cingulate cortex); (5) the descending modulatory neurons that originate in subcortical structures (e.g., the periaqueductal grey and locus ceruleus) and project back to the spinal dorsal horn neurons for descending pain processing [25–27]. 

### 2.2. Central Nociceptive Processing

Modulation of nociceptive signals occurs beyond the peripheral nociceptors in the central nervous system. This modulation includes processes at the spinal cord and at subcortical and cortical brain structures (components 2, 3, 4, and 5 from above). 

First, much of the central nociceptive processing occurs in the spinal dorsal horn [25, 27]. At least two types of spinal secondary neurons are found in the dorsal horn: the nociceptive-specific (NS) neurons and wide dynamic range (WDR) neurons. The WDR neurons are capable of windup, wherein repetitive noxious stimulation with frequencies above 0.3 Hz (the natural frequency of the WDR neurons) leads to amplification in output of the WDR neurons [28]. Such increased wind-up is implicated in a variety of chronic pain conditions [27, 29, 30].

The output of the spinal secondary neurons is dependent on ascending input from the peripheral nociceptors, and it is also modulated by spinal interneurons and descending projections from supraspinal centers. The dynamic balance of these three sources of influence determines the final output from the spinal secondary neurons, which project upward to the relay centers and ultimately to the cerebral cortex for pain perception. This complex interaction of ascending and descending influence on the spinal transmission of pain, commonly referred to as the gate control theory, was originally discovered by Melzack and Wall [31] and has since been validated by many [32, 33].

Second, equally important site of central pain processing occurs in the brain via the complex interaction between the cortex and subcortical nuclei [26, 34–36]. The brain is considered crucial for translating nociceptive signals into the conscious perception of pain. Nociceptive signals are relayed from the thalamus to primary and secondary somatosensory regions, and subsequent brain regions are linked to visceral sensation (i.e., insula), emotion (i.e., limbic system), attention (i.e., anterior cingulate), and cognition (i.e., prefrontal cortex). The brain also exerts descending modulation on nociceptive processing via subcortical structures such as the periaqueductal gray (PAG), the rostroventral medulla (RVM), the hypothalamus, the parabrachial nucleus, and the nucleus tractus solitarius. Complex reciprocal interactions exist between the subcortical and cortical centers of pain processing. Eventually, the descending fibers travel in the dorsal lateral funiculus to reach secondary and inter neurons in the spinal dorsal horn [25]. 

## 3. Dynamic Quantitative Sensory Testing

Quantitative sensory testing (QST), also known as psychophysical testing, refers to tests of sensory perception during the administration of stimuli with predetermined physical properties and following specific protocols [37]. These tests are generally safe and noninvasive for use in human studies, and neuroscience research links these tests to biological underpinnings. Backonja, Arendt-Nielsen, and Pfau [37–39] have published in-depth reviews of quantitative sensory testing. 

QST can be subdivided into static QST and dynamic QST [38, 39]. Static QST typically refers to the measurement of the threshold that primarily reflects states of the peripheral nervous system. Conversely, dynamic QST involves agitation of the pain-perceiving system in a way that exposes a certain mechanism of pain processing beyond the peripheral nervous system. Two extensively studied dynamic paradigms are temporal summation (TS) and conditioned pain modulation (CPM), which represent the ascending facilitatory and descending inhibitory aspects of central pain processing, respectively [38].  Table 1 summarizes the basic concept and characteristics of TS and CPM.

### 3.1. Temporal Summation

Temporal summation (TS) refers to the increased perception of pain in response to repetitive noxious stimuli delivered at frequencies above 0.3 Hz [40, 41]. It is often called “windup pain,” or “temporal summation of second pain.” 

#### 3.1.1. Animal Studies and Molecular Mechanisms

Temporal summation is the behavioral correlate of “windup” of spinal wide dynamic range (WDR) neurons at the dorsal horn [28, 75]. In animal studies, researchers made single-fiber recordings from the periphery C fibers and their destined secondary neurons in the spinal dorsal horn. With successive C-fiber activations (by either noxious heat or noxious electrical stimulation) at frequencies over 0.3 pulses per second, WDR neurons displayed increased frequency and amplitude of discharges [75]. These physiologic changes were correlated to behavioral experiments in humans under the same exact stimulation paradigm: they rated the pain with increasing intensity [28]. Thus, TS QST is thought to represent ascending spinal windup of pain processing. 

#### 3.1.2. Increase of TS in Chronic Pain and Risk Factors

TS is elevated in a wide variety of pain syndromes, ranging from those that cause idiopathic total body pain (e.g., fibromyalgia [76]) to those considered driven entirely by peripheral factors (e.g., knee arthritis [77]). Increasing evidence suggests that abnormally augmented TS is at least partially responsible for the development of these chronic pain conditions [27]. Furthermore, researchers have identified important risk factors (Table 1) that increase TS, including older age [44], female sex [45, 46], psychological factors (anxiety [50], fear [50], and catastrophizing [46–49]), and location of test (the back exhibits higher TS than the upper or lower extremities [50]). 

#### 3.1.3. Methodology for Measurement

Although TS is likely a powerful tool for pain research, the lack of a single, standardized, broadly accepted protocol remains a challenge when interpreting previous work and planning future studies. A variety of noxious stimuli can be used to generate TS, including heat, pin pricks, and electrical stimulation [78]. Although there is no consensus on the quantification of TS [79], 5–20 brief repetitions of identical noxious stimuli are typically given, and the research participant is often asked to rate the changing pain sensation after one or several of the stimuli.  Table 2 outlines examples of several commonly used experimental protocols to generate and compute TS. 

For heat paradigms, the difference in the pain score between the first and most painful pulse, the slope of pain increase, or even the raw pain score from the fifth pulse can be used to calculate the magnitude of TS [42, 61, 79]. When pin pricks are used as the noxious stimuli, the German Research Network on Neuropathic Pain [65] recommends a standard protocol where either 128 or 56 mN pin tips are applied as a single stimulus and as a series of 10 stimuli given at 1 Hz. The participant is asked to give a single pain rating for the single stimulus and then an average rating for the 10 stimuli repeated at 1 Hz. The ratio of average pain rating of the 10 consecutive stimuli to the rating of the single stimulus is defined as TS or, alternatively, as the windup ratio. 

#### 3.1.4. Temporal Summation in Acupuncture Studies

The application of TS to acupuncture research in humans is limited despite the fact that the results of several animal studies indicate that acupuncture produces strong, central modulatory effects and that TS reflects the state of central pain facilitation. Currently, only two clinical studies have been performed involving TS in acupuncture. 

In the first study, Zheng et al. [63] randomized 36 healthy volunteers to blindly receiving 25 minutes of electroacupuncture, manual acupuncture, and nonpenetrating sham acupuncture in one leg. The TS threshold for trains of electrical stimulation was assessed before, immediately after, and 24 hours after the treatments on the ipsilateral leg, contralateral leg, and contralateral arm. The results demonstrated that electroacupuncture increased the TS threshold (i.e., reduced TS) in the ipsilateral and contralateral leg up to 24 hours after the treatment. In contrast, manual acupuncture produced no significant change in the TS threshold, although it showed a trend of increase as compared with sham acupuncture. The increase in TS threshold was the greatest in the ipsilateral leg, followed by the contralateral leg; the least change was seen in the contralateral arm, suggesting segmental specificity of the acupuncture interventions. This is one of the very few studies demonstrating that different forms of needle manipulation produced differences in human pain perception linked to a mechanism of central pain processing. Finally, this study demonstrated peripheral influences of acupuncture, as electroacupuncture augmented not only the TS threshold but also the pain detection threshold to single-pulse electrical stimulation. 

In the second study, by Tobbackx et al. [80], 39 patients with chronic neck pain due to whiplash injury underwent one session of manual acupuncture (20 minutes) and one session of relaxation therapy (length not specified) in random order with a 1-week between-session washout period. The primary outcomes assessed were pressure pain sensitivity to an analogue algometer and TS scores to 10 consecutive applications of pressure stimuli using the same algometer at a pressure above the pain threshold. The study found that acupuncture caused a greater reduction of the pressure pain threshold as compared with relaxation therapy but produced no change in the TS scores. The authors concluded that in patients with chronic pain, acupuncture does not affect central pain processing. 

The inconsistency in the methods used as well as the limited results from these two studies underscore the need for future studies to help further elucidate the role of acupuncture in central pain processing in human subjects. Specifically, both Zheng and Tobbackx demonstrated that after a single session, manual acupuncture did not result in a significant change in TS. However, Zheng was able to demonstrate a significant decrease in TS after electroacupuncture (Tobbackx only studied manual acupuncture). These results suggest that electroacupuncture may exert a stronger influence on TS than manual acupuncture. Future studies should be conducted to compare electroacupuncture with manual acupuncture in larger cohorts of patients with chronic pain.

It is also important to note that these studies involved only one acupuncture session. In acupuncture practice, a single session is rarely considered sufficient to produce clinically significant effects for the treatment of chronic pain. Therefore, when translating the results of studies of animal models and healthy human subjects to the clinical pain population, multiple acupuncture sessions with treatment frequency of at least once a week should be considered. 

We also recommend performing quantitative sensory testing at multiple anatomical sites adjacent to and at a distance from the site treated for pain. Zheng et al. demonstrated a stronger effect of acupuncture in homotopic versus heterotopic sites, while Tobbackx collected data only on the arm, distal to the neck where the pain and the majority of the needling were located. 

Last but not least, there is a need to distinguish the peripheral and central components of acupuncture analgesia. Specifically, Zheng demonstrated increase in the threshold to both temporal summation and to single-pulse protocols, suggesting acupuncture's involvement in both central and peripheral nociceptive modulation. The authors further suggest that a mechanism independent of NMDA blockade, such as peripheral opioid receptor activation [81], may play a role. To test this hypothesis, selective blockade of NMDA and *μ*-opioid receptors should be used. Furthermore, additional biomarkers of central (e.g., secondary hyperalgesia to capsaicin [82]) and peripheral pain processing (pressure and heat pain threshold [38]) may also be used to profile the pain modulatory mechanisms of acupuncture. 

### 3.2. Conditioned Pain Modulation

Conditioned pain modulation (CPM) refers to the phenomenon whereby a noxious stimulus at one body part results in reduced pain perception to another noxious stimulus at a distant, heterotopic body part [83, 84]. The first stimulus is referred to as the conditioning stimulus; the second stimulus, whose pain rating decreases after the application of the conditioning stimulus, is referred to as the test stimulus [84]. CPM has been shown to be a separate phenomenon from cognitive distraction [85, 86]. A variety of other terms have also been used to describe CPM, such as “pain inhibiting pain,” “heterotopic noxious conditioning stimulation,” and “counterirritation [83, 84].” CPM was also referred to as diffuse noxious inhibitory control (DNIC). However, international experts have agreed to distinguish DNIC, a neurophysiologic process, from CPM, a behavioral correlate of this process (see below) [84]. 

#### 3.2.1. Animal Studies and Molecular Mechanisms

CPM is the behavioral correlate to diffuse noxious inhibitory control (DNIC) [84], an inhibitory mechanism involving the spinal-bulbo-spinal loop in animal neurophysiological studies [87]. In 1979, Le Bars et al. discovered that when noxious stimuli (A-*δ*- or C-fiber-mediated) are applied anywhere outside the excitatory receptive field of a spinal or trigeminal WDR neuron, the response to any noxious input within its receptive field is profoundly inhibited [88, 89]. Le Bars' group subsequently found that DNIC is mediated by the subnucleus reticularis dorsalis (SRD) in the caudal medulla [90], which receives noxious input via pathways in the ventral lateral quadrant of the spinal cord [91], and sends global inhibitory signals to WDR neurons from all spinal levels via the dorsolateral funiculi [92]. Finally, the strong correlation between the signal reduction in the WDR neurons and the pain reduction in a CPM paradigm, in both extent and time course, supports the notion that CPM is the behavioral correlate of DNIC [93–95].

#### 3.2.2. Decrease of CPM in Chronic Pain and Risk Factors

Like TS, CPM is altered in many chronic pain conditions, such as fibromyalgia [96], tension-type headache [97], irritable bowel syndrome [98], and arthritis of the hip [99]. Rather than an increase as with TS, a decrease in CPM is seen. As shown in Table 1, the risk factors for decreased CPM are similar to those for increased TS. These include older age [44, 51, 52], female sex [43, 45, 53], and psychological factors such as catastrophizing [54, 55]. However, the relationship between female sex and decreased CPM is less straight forward as some studies showed clear increase in CPM associated with female sex, while others did not find such association [43, 45, 53]. Large variations in methodology may partially contribute to this discrepancy [43, 53]. Furthermore, chronic opioid use [59], depressed mood [58], and poor sleep also decrease CPM [56, 57]. 

#### 3.2.3. Measurement Methodology

There is no single, standard protocol for measuring CPM.  Table 3 summarizes the key components in generating CPM and demonstrates examples of their variability. Pud et al. [43] published an excellent review of CPM methods. In short, a test stimulus is measured before the application of the conditioning stimulus to obtain a baseline and is measured again during or after the application of the conditioning stimulus to quantify the magnitude of CPM relative to the baseline. The noxious test and conditioning stimuli are typically administered at anatomically distinct locations.

The most common conditioning stimulus is a cold water bath applied to the contralateral hand. However, other conditioning stimuli have been used, including isotonic saline injections and heat pain. It is the general consensus that the conditioning stimulus must be noxious in order to trigger CPM [43, 53]. Once the noxious threshold has been exceeded, the intensity of the conditioning stimulus may not matter, according to reports by Granot et al. [68] and Nir et al. [100]. The duration of the conditioning stimulus is usually between 30 seconds and 2 minutes for the cold water bath [53].

In contrast to the conditioning stimulus, there is a large variation in the choice of test stimulus. Pain recordings of a standard stimulus or pain thresholds from any type of stimulation (electrical, chemical, heat, pressure, etc.) can be used [43]. The magnitude of CPM is measured by the change from baseline in the test stimulus to after the conditioning stimulus is applied. The CPM effect appears to peak during the application of the conditioning stimulus and fades rapidly from 5 to 10 minutes after the conditioning stimulus ceases [43, 53, 101, 102]. One report indicates that the approximate median magnitude of CPM represents about a 29% decrease in pain rating, regardless of the test stimulus used [43]. There is some indication that CPM is stronger when the test and conditioning stimuli are applied at a greater anatomical distance from the CPM stimulus site (e.g., CPM is stronger for the hand-to-contralateral leg than for the hand-to-contralateral hand) [43, 87].

In summary, the best means of capturing robust CPM is to use a strong, noxious conditioning stimulus (such as cold immersion of the contralateral distal extremity) and measure the change in the test stimulus during the latter part of the conditioning stimulus. As with TS, significant variations exist in the methodologies used to generate and compute CPM, making it difficult to make comparisons across studies and subjects. Future efforts should focus on identifying a standardized, broadly accepted protocol for CPM. 

#### 3.2.4. CPM in Acupuncture Studies

Similar to TS, the use of CPM to study acupuncture analgesia is limited. There are only two direct studies on acupuncture analgesia and CPM/DNIC, both of which focused on the question of whether acupuncture analgesia is equivalent to CPM/DNIC. To date, no one has studied how acupuncture stimulation may modulate the extent of DNIC. 

The first acupuncture-DNIC study was carried out by Bing et al. [103]. Output from WDR neurons in the trigeminal nucleus of rodents was measured using the patch-clamp technique. The conditioning stimuli consisted of manual acupuncture applied to Zusanli (a classic acupuncture point) and to an adjacent nonacupuncture point, both located on the right hind limb, and a standard noxious stimulus—immersion of the left hind limb in a 48°C hot water bath. All three stimuli resulted in a similar degree of inhibition in the firing of the trigeminal WDR neurons (72.5% and 78.5%). Furthermore, the inhibition resulting from all three stimuli demonstrated a similar time course for decay and a similar response to naloxone, which reversed the inhibition by about 30%. The authors concluded that acupuncture maneuvers trigger the neural mechanisms involved in DNIC. 

The second acupuncture-DNIC (CPM) study was done in healthy human volunteers using a crossover design [104]. It directly compared the effects of acupuncture, sham acupuncture, and a 1.5°C cold water bath (as a conditioning stimulus in the upper extremity). The test stimulus was the pressure pain threshold at the second toe. The verum acupuncture involved the penetration of Hegu (a classic acupuncture point) with a needle without manipulation, and retaining the needle for 5 minutes. The sham acupuncture involved the tapping and placement of a nonpenetrating Streitberger device [105] at Hegu for 5 minutes. The results showed that the cold bath resulted in much stronger increase in the pressure pain threshold as compared with verum and sham acupuncture. Furthermore, there was no statistically significant difference between the verum and sham. The authors concluded that acupuncture as performed in this trial exerted a small analgesic effect not different from placebo and that the analgesic effect was unlike CPM. 

It is difficult to compare these studies because of several reasons. First, the test and conditioning stimuli differed significantly between the two studies. Second, as the authors of the second paper mentioned, their acupuncture needling was only minimally painful (pain score about 2.4 ± 1.5 out of a 10-point scale). Because prior studies have shown that CPM will only work when the conditioning stimulus is beyond the noxious threshold, it is not surprising that acupuncture did not trigger CPM in these studies. Third, Deqi, a unique composite of sensations (such as deep ache and tingling sensation) considered essential for clinical efficacy according to traditional Chinese medicine [106], was not elicited in the second study. In real clinical practice, the acupuncturist aims to achieve Deqi, retains the needles in the body for between 15 and 20 minutes, and often uses more than one needle [107]. Therefore, the treatment performed in the second study does not represent typical clinical practice. Future studies should focus on adapting CPM for the clinical study of acupuncture analgesia using acupuncture methods that are consistent with clinical practice.

Last but not least, the duration of CPM/DNIC is short-lived. Directly comparing acupuncture with CPM will not help understand the clinically relevant long-term analgesia by acupuncture. Because DNIC/CPM is considered to play a role in mediating pain perception in chronic pain conditions [27, 108, 109], it would be more relevant to study how acupuncture influences CPM rather than simply to treat acupuncture as a form of transient conditioning stimulus. 

### 3.3. Current Trends and Future Directions QST in Acupuncture Research

#### 3.3.1. Clinical Applications of QST

Quantitative sensory testing is increasingly used in clinical research as a helpful marker for central and peripheral nociceptive processing [38, 39, 110]. Specifically, researchers are applying TS and CPM to the following three categories of translational research: (1) to phenotype patient subgroups based on different underlying pain mechanisms [27, 53, 109, 111]; (2) to predict response to treatment based on mechanistic phenotypes thus determined [112–116]; and (3) to characterize the central modulatory effects of new therapies [99, 117–121]. For example, as mentioned separately in the TS and CPM sections, many chronic pain conditions display increased TS and/or reduced CPM as compared with healthy controls. These conditions include fibromyalgia, TMJ disorder, headaches, irritable bowel syndrome, back pain, and arthritis of hip and knee [27]. The next step, then, is to select the appropriate therapies that specifically address an individual patient's mechanistic deficit(s). Recently, Yarnitsky et al. demonstrated this concept of mechanism-based treatment of pain in a landmark study [116]. He found that deficient descending inhibition, as reflected by low CPM, predicted clinical response to duloxetine, an antidepressant that augments descending inhibition by increasing serotonin and norepinephrine levels in the central nervous system [122]. This study represents a future of personalized pain treatment, where QST fingerprinting provides key information on the mechanism of an individual's pain condition. Last but not least, TS and CPM have been used to characterize the central modulatory effects of many drugs and interventions, such as ketamine [123], gabapentin [117], surgery [120], and exercise [121]. 

#### 3.3.2. Limitations of Current QST Research

Despite the advances described here, there are still significant methodological barriers to the wide utilization of QST in clinical research. First, although the temporal stability of TS and CPM has been established in healthy volunteers [42, 66, 124], it has not been characterized in chronic pain patients [110]. Without stability data in this population, it is difficult to discern if a change in TS or CPM is due to the therapeutic intervention or to the natural fluctuation in these measures. Second, a variety of methods have been used to generate and compute TS [42, 79] and CPM [43], which makes it challenging to make comparisons across studies. Third, individual TS and CPM responses vary widely. For example, a standard thermal TS protocol may not capture TS from 40% to 60% of the individuals tested [61, 125]. This variability imposes restrictions on the external validity of studies involving TS and CPM, particularly in longitudinal studies where some degrees of TS and CPM are expected at baseline. Our group is actively searching for solution to all 3 issues by assessing the stability of TS and CPM in patients with chronic pain, developing universal protocols to capture TS and CPM in most individuals, and investigating the physiologic bases of the between-individual variations in the response to TS and CPM tests. 

In summary, while there is a large body of literature on using TS and CPM to study chronic pain, limitations in methodology still exist. Future studies should address the following three issues: (1) the uncertainty in the temporal stability of TS and CPM in chronic pain conditions; (2) the lack of a universal protocol; (3) the large between-individual variability in both TS and CPM.

#### 3.3.3. QST in Acupuncture Research

The literature contains only sparse data on the use of QST in human acupuncture research. This may be due, in part, to lack of awareness. Advances are being made in applying QST to understand the mechanisms of chronic pain [27, 38, 53, 78, 109] and medications [29, 112, 116–118, 126], and we believe there is a definitive role for QST in the study of acupuncture analgesia. 

We make the following practical recommendations for utilizing QST in clinical acupuncture studies. First, because the stability of TS and CPM has not been established in the clinical pain population, we recommend a parallel group design with one group representing the natural course of the disease (waitlist control) and other groups representing active and sham interventions. Second, although there is no single standardized protocol for TS or CPM, there are both more and less established protocols. We recommend choosing the more established protocols. For example, the OPERA trial of several thousands of patients with oral facial pain used reliable heat and pressure pain paradigms to examine TS and CPM [111]. Another example is the comprehensive set of QST measures that encompass both the dynamic and static measures developed by the German Research Network in Neuropathic Pain (DFNS) [65]. With a strong focus on peripheral pathologies, this protocol has been validated on a variety of neuropathic pain syndromes and has been shown to be consistent [127]. Furthermore, both the OPERA and the DFNS protocols include traditional threshold measures to a variety of sensory stimuli. Unlike CPM and TS, these threshold measures offer additional insights on peripheral nociceptive processes. Last but not least, we recommend a two-step approach to determine whether acupuncture leads to analgesia by modifying the central pain-processing pathways. First, research should test whether acupuncture will lead to changes in dynamic QST parameters such as TS and CPM. Second, studies should investigate whether such changes lead to improvement of pain and function. 

## 4. Conclusions

Numerous animal studies suggest that acupuncture leads to analgesia via powerful central pain modulatory mechanisms. However, little is known about whether and how these findings may translate to clinically meaningful outcomes. TS and CPM are emerging behavioral correlates of ascending excitatory and descending inhibitory limbs of central pain modulation. Both TS and CPM have been widely used in clinical pain research, yet their application to the understanding of acupuncture analgesia is limited. We encourage greater use of TS and CPM in acupuncture research, in conjunction with other psychophysical tools such as pain detection thresholds, and with appropriate attention given to the limitations of these psychophysical methods. The appropriate adaption of dynamic (TS and CPM) and static QST measures (pain detection threshold) will help advance our understanding of the true mechanisms of acupuncture analgesia in human clinical populations.

## Figures and Tables

**Table 1:** Overview of TS and CPM.

	TS	CPM
Experimental construct	Repeats of brief noxious stimuli	A test stimulus measured before and after a conditioning stimulus
Typical magnitudes in healthy subjects	10–20 in a 0–100 visual analog scale (VAS) [42]	~29% reduction in pain rating [43]
Underlying CNS physiology	Windup: increased spinal WDR output due to repetitive C-fiber stimulation at >0.3 Hz	DNIC: global reduction of WDR sensitivity due to a single, heterotopic, noxious stimulation
Pain-processing pathways involved	Ascending facilitation of nociceptive input	Descending inhibition of nociceptive input
Augmenting factors	Advanced age [44], female sex [45, 46], pain catastrophizing [46–49], anxiety, fear of pain, and location (trunk > extremities) [50]	
Reducing factors		Advanced age [44, 51, 52], female sex (mixed results [43, 45, 53]), pain catastrophizing [54, 55], poor sleep [56, 57], depression [58], and opioid use [59]

**Table 2:** Common methods used to generate and compute TS.

Type of stimulus	Experimental paradigms	Variables used to quantify TS
Heat pulses	10–20 heat pulses (0.5–0.75 s each) delivered at 0.3–0.5 Hz either via a continuous contact thermode [44] or intermittent contact probe [60]	TS magnitude: the difference in pain ratings between first and last, or first and most painful pulse, slope of the first few pulses, or the magnitude of 5th pulse [42, 61]
Electrical stimulation	A single stimulus of a train of five 1-ms pulses at 200 Hz, repeated 5 times at 2 or 3 Hz [62, 63]	Electrical pain threshold (EP-T): intensity at which the subject begins to feel pain at the 4th or 5th pulse [62, 63], or nociceptive withdrawal reflex threshold (NWR-T), the intensity at which limb flexion occurs [64] in response to the electrical stimulation
Pin prick	10 stimuli of 56 or 128 mN are delivered, and pain ratings for all ten stimuli averaged versus that of a single stimulus are obtained [65]	Windup ratio: pain of train of 10 pricks delivered at 1 Hz over pain of a single prick [65]
Pressure	Ten 1-s pressure stimuli delivered by an algometer with 1 s between pulses [66, 67]	TS magnitude: difference in pain rating between the first and 10th stimuli [66, 67]

**Table 3:** Examples of varied parameters in generating CPM.

Parameter	Examples
Conditioning stimulus	Cold water bath (0–10°C) [68], heat (thermode or water bath) [69], hypertonic saline injection [70], and inflated blood pressure cuff [71]
Testing stimulus	Pain detection thresholds [69], rating of a predetermined single pain stimulus [68], and TS protocols [72]
When to measure test stimulus again	Varies widely: from during the conditioning stimulus [73] up to 30 min after the conditioning stimulus [74]
Location of stimulus	Large variation in the relative distance between the testing and conditioning stimuli: for example, upper body to upper body [68] versus upper body to lower body [51]
Computation	Relative or absolute changes in threshold measures or ratings of predetermined pain stimulus [43]

## Abbreviations

AA: Acupuncture analgesia
TS: Temporal summation
CPM: Conditioned pain modulation
QST: Quantitative sensory testing
DNIC: Diffuse noxious inhibitory control
NS: Nociceptive specific
WDR: Wide dynamic range
PAG: Periaqueductal gray
RVM: Rostroventral medulla
SDR: Subnucleus dorsalis reticularis
S1: Somatosensory area 1
S2: Somatosensory area 2.
